# Anti-Respiratory Syncytial Virus Activity of *Plantago asiatica* and *Clerodendrum trichotomum* Extracts In Vitro and In Vivo

**DOI:** 10.3390/v11070604

**Published:** 2019-07-03

**Authors:** Kiramage Chathuranga, Myun Soo Kim, Hyun-Cheol Lee, Tae-Hwan Kim, Jae-Hoon Kim, W. A. Gayan Chathuranga, Pathum Ekanayaka, H. M. S. M. Wijerathne, Won-Kyung Cho, Hong Ik Kim, Jin Yeul Ma, Jong-Soo Lee

**Affiliations:** 1 Microbiology Laboratory, Department of Preventive Veterinary Medicine, College of Veterinary Medicine, Chungnam National University, Daejeon 34134, Korea; 2 Vitabio Corporation, Daejeon 34540, Korea; 3 Laboratory Animal Resource Center, Korea Research Institute of Bioscience and Biotechnology, University of Science and Technology (UST), Daejeon 34141, Korea; 4 Korean Medicine (KM) Application Center, Korea Institute of Oriental Medicine, Daegu 41062, Korea

**Keywords:** *Plantago asiatica*, *Clerodendrum trichotomum*, RSV, therapeutic effects, acteoside

## Abstract

The herbs *Plantago asiatica* and *Clerodendrum trichotomum* have been commonly used for centuries in indigenous and folk medicine in tropical and subtropical regions of the world. In this study, we show that extracts from these herbs have antiviral effects against the respiratory syncytial virus (RSV) in vitro cell cultures and an in vivo mouse model. Treatment of HEp2 cells and A549 cells with a non-cytotoxic concentration of *Plantago asiatica* or *Clerodendrum trichotomum* extract significantly reduced RSV replication, RSV-induced cell death, RSV gene transcription, RSV protein synthesis, and also blocked syncytia formation. Interestingly, oral inoculation with each herb extract significantly improved viral clearance in the lungs of BALB/c mice. Based on reported information and a high-performance liquid chromatography (HPLC) analysis, the phenolic glycoside acteoside was identified as an active chemical component of both herb extracts. An effective dose of acteoside exhibited similar antiviral effects as each herb extract against RSV in vitro and in vivo. Collectively, these results suggest that extracts of *Plantago asiatica* and *Clerodendrum trichotomum* could provide a potent natural source of an antiviral drug candidate against RSV infection.

## 1. Introduction

Acute respiratory infections caused by viruses are the most common cause of morbidity and mortality in children worldwide. Respiratory syncytial virus (RSV) is one of the major causes of lower respiratory tract infections, which cause a huge global disease burden [1]. It is the most important viral agent of serious respiratory tract illness in infants and young children. Nearly all infants have been infected with RSV at least once by the age of two years [2]. RSV is also a major cause of acute respiratory illness in the elderly, and it can have a detrimental effect in immune-compromised individuals. Even though RSV infection generally occurs at an early age, individuals may be re-infected throughout their lifetime, because naturally acquired immunity does not provide persistent protection [3]. At present, RSV vaccines and antiviral drugs are in the preclinical and clinical stage of development; however, no RSV vaccines or antiviral drugs suitable for typical use are commercially available at this time [4]. Ribavirin and immunoglobulin preparations with high titers of RSV-specific neutralizing antibodies are currently approved to treat and prevent RSV infections [5]. However, neither of these options is cost-effective or convenient to administer. Due to the high infant morbidity and mortality rates, the lack of an effective vaccine, and the availability of just one antiviral agent (Ribavirin), which is used only in severe cases, novel therapies for RSV infection warrant investigation.

Fossil evidence has revealed that human use of plants as folk medicine dates back at least 60,000 years [6]. According to the World Health Organization (WHO), almost 65% of the world’s population reports the use of natural compounds as medicinal agents [7]. Modern analytical technologies applied towards the active compounds found in plants have allowed for greater insights into plant-derived pharmaceutical compounds [8]. *Plantago asiatica* is a perennial belonging to the family Plantaginaceae and is commonly used as a folk medicine in Korea, China, and Japan [9]. *Plantago asiatica* extract (PAE) has been used to treat a variety of health conditions, such as wounds, cholesterolemia, diarrhea, bronchitis, and chronic constipation [10,11,12]. Moreover, it has been shown to inhibit cancer and leukemia growth and to enhance cell-mediated immunity [13]. *Clerodendrum trichotomum* is a deciduous shrub that belongs to the family Lamiaceae (formerly Verbenaceae) and is widely distributed in South Korea, China, Japan, and the Philippines. *Clerodendrum trichotomum* extract (CTE) possesses broad-spectrum anti-inflammatory [14,15], anti-hypertensive [16], anti-asthmatic [17], anti-oxidative [18] and immunotoxin [19] properties. However, the effects of PAE and CTE and their active components on RSV replication in vitro and in vivo have not been reported.

In this study, we evaluated the antiviral activities of *P. asiatica* and *C. trichotomum* aqueous extracts against RSV in vitro and in vivo. Furthermore, we identified and confirmed the antiviral function of acteoside (verbascoside), a phenolic glycoside presents in both aqueous extracts, as the potential active component with antiviral activity against RSV infection.

## 2. Materials and Methods

### 2.1. Plant Materials and Total Aqueous Extract Preparation

A water-soluble extract of *P. asiatica* and *C. trichotomum* was prepared by Herbal Medicine Improvement Research Center, Korea Institute of Oriental Medicine, Daejeon, Republic of Korea. Crude plant materials were purchased from a local store (Jaecheon Oriental Herbal Market) and verified by Professor Ki-Hwan Bae at the College of Pharmacy, Chungnam National University. In the proses, 100 g of the plant materials were placed in 1 L of distilled water and extracted by heating for 2.5 h at 105 °C using a medical heating plate. After the extraction proses, the extract was subjected to filtration using a filter paper (0.45 μm, Millex®, Darmstadt, Germany) and stored at 4 °C for 24 h. The extract was then centrifuged at 8000× *g* for 15 min. The supernatant was collected, and the pH was adjusted to 7.0. Following pH adjustment total successive aqueous extract was subjected to membrane syringe filtration (0.22 μm) and stored at −20 °C until further use.

### 2.2. Reagents, Chemicals and Antibodies

Verbascoside (Acteoside) was purchased from Sigma (V4015). Trypan blue solution was purchased from Gibco (Waltham, MA, USA). Cell cytotoxicity assay kit was purchased from Dojindo Molecular Technologies, INC (CK04: Cell Counting Kit-8, Japan). Antibodies used in the immunoblotting study were as follows: Anti-RSV Glycoprotein (RSV-G) (Abcam, #ab94966, Cambridge, UK), β-actin (Santa Cruz SC 47778, Dallas, TX, USA), HRP-conjugated anti-mouse IgG (Gene Tex, GTX213111-01, Taichung, Taiwan), HRP-conjugated anti-rabbit IgG (Cell signaling technology, 7074P2, Danvers, MA, USA).

### 2.3. Cell Culture and Virus Infection

Human epithelial type *2*: HEp2 cells with HeLa contaminant (ATCC CCL-23, Manassas, VA, USA) and A549 cells (ATCC CCL-185, Manassas, VA, USA) were maintained in Dulbecco’s Modified Eagle’s Medium (DMEM) (Invitrogen, Waltham, MA, USA) supplemented with 10% fetal bovine serum (FBS) (Hyclone, Australia) and 1% antibiotic/antimycotic solution (Gibco, Waltham, MA, USA) at 37 °C with a 5% CO_2_ environment. The Green Fluorescent Protein fused Respiratory syncytial virus (RSV-GFP) from Dr. Jae U. Jung, Department of Molecular Microbiology and Immunology, University of Southern California, USA. RSV-GFP propagated on confluent HEp2 cells, and titer was determined by a standard plaque assay.

### 2.4. Antiviral Assays

RSV-GFP virus replication inhibition assay was performed in vitro using HEp2 cells, as described previously with minor modifications [20]. Briefly, HEp2 cells and A549 cells were seeded in 12 well cell culture plates with the cell number of 2.5 × 10^5^ cells/well and incubated for 12 h. Medium was changed with DMEM containing 1% FBS and Cells were infected with RSV-GFP [multiplicity of infection (MOI) 0.1] for 2 h. Cells were washed with PBS and medium was replace with DMEM containing 10% FBS and cells were treated with indicated concentrations of PAE, CTE or acteoside. GFP expression was measured at 48 h post infection (hpi) with Glomax multidetection system following manufacturer’s directions. Virus titer was determined in supernatant and cells by plaque assay in HEp2 cells or A549 cells [21]. Cell viability was evaluated using a trypan blue exclusion test as described previously [22].

### 2.5. Determination of Effective Concentration (EC_50_) of Extracts and Acteoside

HEp2 cells were grown in 24-well cell culture plates (1.25 × 10^5^ cells/well) and incubated at 37 °C in a 5% CO_2_ atmosphere. After 12 h, the medium was replaced with DMEM containing 1% FBS and cells were infected with RSV-GFP (0.1 MOI) for 2 h. Then cells were washed with PBS once, and the medium was replaced with DMEM containing 10% FBS. Cells were treated with indicated concentrations of herb extracts or acteoside. The experiment was performed in triplicate. GFP expression was measured 48 hpi with the Glomax multi-detection system (Promega, Fitchburg, WI, USA) according to the manufacturer’s instructions. The EC_50_ values were then calculated as the extract concentration yielding 50% GFP expression.

### 2.6. Determination of Cytotoxic Concentration (CC_50_) of Extracts and Acteoside

Cell cytotoxic concentration of herb extracts and acteoside was determined using Cell counting kit-8 Dojindo Molecular Technologies, as described previously [23]. Briefly, HEp2 cells were seeded into 96-well cell culture plates (2.5 × 10^4^ cells/well) and incubated for 12 h. Cells were treated with indicated concentrations of herb extracts or acteoside. Next, at 48-h post treatment, 10 μL of CCK-8 solution was added to each well of the plate. Then, it was incubated for 1 h at 37 °C, and absorbance was measured at 450 nm using microplate reader (molecular devices).

### 2.7. Quantitative RT-PCR (qRT PCR)

Total RNA was extracted from cells, or 1 g of lung homogenate using the RNeasy Mini kit (Qiagen, Hiden, Germany) and cDNA synthesis was performed using the enzyme reverse transcriptase (TOYOBO). Next, qRT-PCR was performed using the Rotor Gene Q instrument (Qiagen, Hiden, Germany), with the QuantiTect SYBR Green PCR Master Mix (Qiagen, Hiden, Germany). The transcription level of mRNA was obtained by the 2^−ΔΔ*C*t^ method as described previously [24] and expressed as fold induction. The RT-PCR primer sequences used as follows, RSV-G forward primer 5’-CCAAACAAACCCAATAATGATTT-3’ reverse primer 5’-GCCCAGCAGGTTGGATTGT-3’ Glyceraldehyde 3-phosphate dehydrogenase (GAPDH): Forward primer 5’-TGACCACAGTCCATGCCATC-3’ reverse primer 5’-GACGGACACATTGGGGGTAG-3’.

### 2.8. Immunoblot Analysis

HEp2 cells were seeded in six well cell culture plates (5 × 10^5^ cells/well) and incubated for 12 h. Medium was changed to DMEM containing 1% FBS and cells were infected with RSV-GFP (0.1 MOI) for 2 h. Cells were treated with PAE, CTE or acteoside once replacing the medium with DMEM containing 10% FBS. Cells were harvested at 0, 12, 24, 36, 48, hpi and subjected to immunoblot analysis. Briefly, harvested cells were lysed with lysis buffer containing 1% NP-40, 150 mM NaCl, 50 mM Tris-HCl pH 8.0 and a protease inhibitor (Sigma). Whole cell lysates were mixed with 10x sample buffer (Sigma, St. Louis, MO, USA) at 1:1 ratio, and the total protein was separated in 12% gel by SDS-PAGE and transferred to a PVDF membrane (BioRad, Hercules, CA, USA). The membrane was blocked in 5% bovine serum albumin (BSA, Sigma) and incubated with anti RSV-G antibody or anti-β-actin antibody with 5% BSA and TBST (Tris-buffered saline (LPS Solution) + Tween 20 (Life science, #0777-1L, Suwanee, GA, USA)) respectively. Proteins were detected by incubating with a secondary anti-rabbit IgG-HRP or anti-mouse IgG-HRP for 1 h at room temperature. The membrane was developed with ECL reagent mix (LPS solution, FEMTO-100) and images were captured with an Enhanced Chemiluminescence Detection (ECL) system (GE Life science, Pittsburgh, PA, USA), using Las-4000 mini lumino-image analyzer (GE Life Science). Band intensity was calculated using ImageQuant LAS 4000 control software (Pittsburgh, PA, USA).

### 2.9. Syncytium Formation Assay

The ability of the herb extract and acteoside to block cell to cell spread was evaluated using GFP expression in the cells. HEp2 cells were cultured in 12 well cell culture plates (2.5 × 10^5^ cells/well) and incubate for 12 h. Medium was changed to DMEM containing 1% FBS and cells were infected with RSV-GFP (0.1 MOI) for 2 h. Cells were treated with PAE/CTE or acteoside once replacing the medium with DMEM containing 10% FBS. After 48 h incubation cells were washed with cooled PBS and cell images were taken under 400 magnifications. Syncytium formation was quantified with imageJ 1.48 program (https://imagej.net/ImageJ).

### 2.10. RSV-GFP Challenge Experiment in Mouse Model

Five-week-old female BALB/c mice were purchased from orient bio (South Korea) and acclimated for three days under experimental condition prior to use. Mice were separated into experimental groups as virus infected, and herb or acteoside treated group (*n* = 5), virus only infected group (*n* = 5) and un infected group (*n* = 2). Mice were anesthetized with ketamine for a short time period, and RSV-GFP 1 × 10^6^ Plaque forming unit (PFU) per mice in the total volume 28 μL was infected intranasally. Mice were orally administered 0.5 mg/mL concentration of PAE or CTE at a total volume of 200 μL at 6, 12, 18 and 24 hpi. In the case of acteoside, 80 mg/Kg of body weight/mice was intraperitonially administered to mice at 6 hpi in the total volume of 100 μL. Lung tissues from euthanized mice were collected aseptically at 3- and 5-day post infection (dpi). Lung RSV titration was determined by RSV-G protein mRNA transcription fold quantification. RSV-G protein mRNA level was quantified as described before.

### 2.11. Ethical Approval

The animal study was conducted under appropriate conditions with the approval of the Institutional Animal Care and Use Committee of Chungnam National University (Reference number CNU-00816).

### 2.12. Identification of Acteoside through HPLC

A reversed-phase high-performance liquid chromatography (HPLC) was performed using Agilent technologies 1200 series HPLC system equipped with a DAD detector (Agilent technologies, Santa Clara, CA, USA). The binary mobile phase consisted of water containing 1% formic acid (solvent A) and acetonitrile (solvent B). All solvents were filtered through a 0.45 µm filter prior to use. The mobile phase consisted of 1% Formic acid (Solvent A) and Acetonitrile (Solvent B) in the gradient mode as follows: 0–20 min 0–40% B; 20–22 min 40–100% B; 22–25 min 100–0% B at flow rate of 1.0 mL/min at 30 °C.

### 2.13. Statistical Analysis

Data are presented as the means ± standard deviations (SD) and are representative of at least three independent experiments. Graphs and all Statistical analysis were performed using GraphPad Prism software version 6 (San Diego, CA, USA) for Windows. Differences between untreated and herb or acteoside treated groups were analyzed by Unpaired *t*-test. *p* < 0.05, *p* < 0.01 or *p* < 0.001 was regarded as significant.

## 3. Results

### 3.1. Antiviral Effects of PAE and CTE

**Figure 1:**
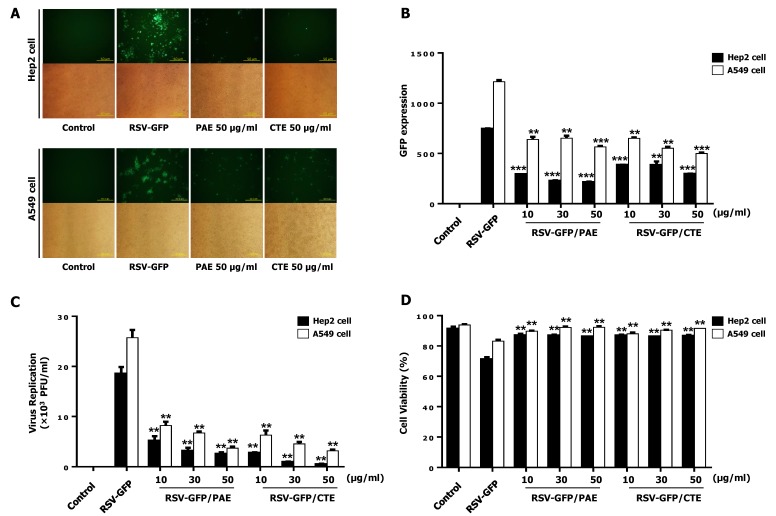
Antiviral activity of *Plantago asiatica* extract (PAE) and *Clerodendrum trichotomum* extract (CTE) in HEp2 cells and A549 cells. HEp2 cells and A549 cells were seeded into 12 well cell culture plates with the cell number of 2.5 × 10^5^ cells/well. Twelve hours later, the medium was changed to 1% fetal bovine serum (FBS) containing Dulbecco’s Modified Eagle’s Medium (DMEM) and cells were infected with Green Fluorescent Protein fused Respiratory syncytial virus (RSV-GFP) 0.1multiplicity of infection (MOI) or kept uninfected. Two hours later, the medium was replaced with 10% FBS containing DMEM and cells were treated with 10, 30, 50 (μg/mL) PAE or CTE. Cells without any treatment regard as virus only. (**A**) After 48 h, images were obtained (200× magnification). (**B**) GFP absorbance levels were measured by Gloma multi-detection luminometer (Promega). (**C**) Virus titration was done from the cell supernatant and cells by standard plaque assay and expressed as plaque forming unit (PFU). (**D**) Cell viability was determined by trypan blue exclusion assay at 48hour post infection (hpi). GFP absorbance, cell viability and virus titer expressed as mean ± standard deviations (SD). Error bars indicate the range of values obtained from counting duplicate in three independent experiments (** *p* < 0.01 and *** *p* < 0.001 regarded as significant difference).

A library of herb extracts was screened to detect antiviral activity against RSV. Among them, PAE and CTE were selected. The ability of the two herb extracts to inhibit the replication of GFP-tagged RSV (RSV-GFP) was further confirmed in a dose-dependent experiment. The expression of RSV-GFP, the virus titer, and the recovery of RSV-induced cell death were evaluated in HEp2 cells upon herb treatment. As shown in Figure 1A,B, HEp2 cells and A549 cells treated with PAE and CTE (10, 30, or 50 μg/mL) exhibited a marked reduction in GFP expression compared to untreated HEp2 cells and A549 cells. Moreover, all doses of the two-herb extract significantly reduced the RSV titer compared to the untreated group (Figure 1C). Interestingly, treatment with PAE and CTE significantly reduced RSV-induced HEp2 cell and A549 cell death at 48 hpi (Figure 1D). Therefore, both herb extracts could significantly reduce RSV replication in HEp2 cells and A549 cells. Since the 50 μg/mL concentration was the most effective at inhibiting viral replication and virus-induced cytotoxicity, this concentration was used for further in vitro experiments.

### 3.2. Therapeutic Effect of PAE and CTE against RSV Infection

**Figure 2:**
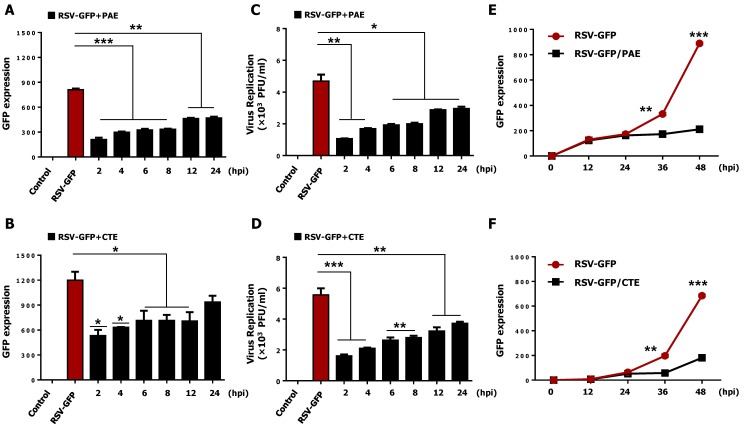
The therapeutic effect of PAE and CTE against RSV-GFP infection. HEp2 cells were seeded into 12 well cell culture plates and left for 12 h. Medium was changed with DMEM containing 1% FBS and cells were infected with RSV-GFP (0.1MOI) for 2 h. (**A**,**B**) RSV-GFP infected cells were treated with 50 μg/mL PAE or CTE at different times after post infection as indicated or left untreated, and GFP expression level was measured at 48 hpi. (**C**,**D**) Virus titer was measured from both supernatant and cells by standard plaque assay at 48 hpi and expressed as PFU. (**E**,**F**) Cells were treated with 50 μg/mL PAE or CTE, and GFP expression level was measured at different time after virus infection as indicated. GFP absorbance and virus titer expressed as mean ± SD. Error bars indicate the range of values obtained from counting duplicate in three independent experiments (* *p* < 0.05, ** *p* < 0.01 and *** *p* < 0.001 regarded as significant difference).

The ability of PAE and CTE to inhibit virus replication after the infection was determined. HEp2 cells were infected with RSV-GFP 0.1 MOI, and herb extracts were added at the indicated time points. GFP expression was measured at 48 hpi. As expected, increased GFP expression at 48 hpi was observed as the amount of time between viral infection and herb treatment increased (Figure 2A,B). Similarly, RSV titers increased with increasing time between viral infection and herb extract treatment (Figure 2C,D). Next, to assess the effect of PAE and CTE on virus replication, an assay was performed, and GFP expression was measured at different times after virus infection. As shown in Figure 2E,F, a 50 μg/mL concentration of each herb extract significantly reduced the GFP expression at 36 hpi and 48 hpi but, interestingly, not at 12 hpi or 24 hpi.

### 3.3. Synergistic Anti-RSV Effect of PAE and CTE in HEp2 Cells

**Figure 3:**
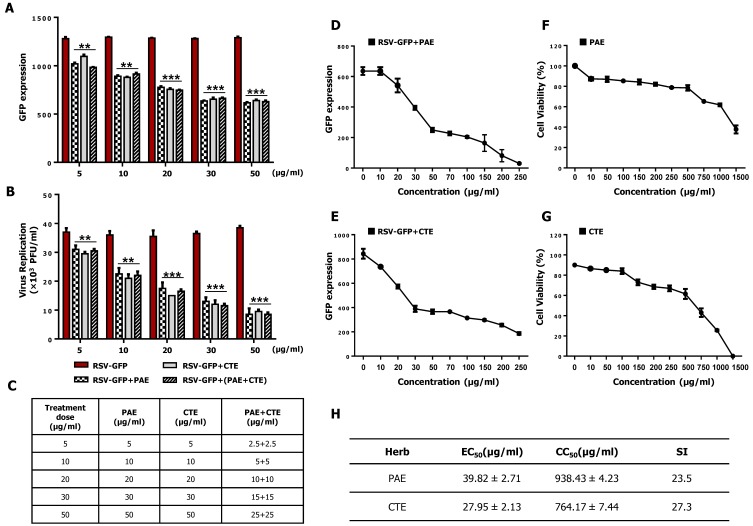
Synergistic effect and effective concentration (EC_50_), cytotoxic concentration (CC_50_) of PAE and CTE in HEp2 cells. (**A**) HEp2 cells were infected with RSV-GFP (0.1 MOI) for 2 h with DMEM containing 1% FBS. Cells were treated with PAE, CTE or combination of both at different concentrations with DMEM containing 10% FBS. At 48 hpi GFP expression level was determined. (**B**) Virus titer was measured from both supernatant and cells by standard plaque assay at 48 hpi and expressed as PFU. (**C**) Dose information of PAE and CTE single or combination treatment. (**D**,**E**) HEp2 cells were infected with RSV-GFP (O.1MOI) for 2 h with DMEM containing 1% FBS. Then, the medium was changed to DMEM containing 10% FBS and cells were treated with various concentrations of PAE (**D**) or CTE (**E**). 48 hpi GFP expression level was determined. (**F**,**G**) HEp2 cells were treated with various concentrations of PAE (**F**) or CTE (**G**), and cell viability was determined at 48 h post treatment (hpt) by cell cytotoxicity assay kit. (**H**) To calculate EC_50_ value, 50% reduction of GFP expression was considered as equivalent to the 50% reduction in virus titer. The ratio between CC_50_ and EC_50_ was considered as Selectivity Index (SI). GFP absorbance and cell viability expressed as mean ± SD. Error bars indicate the range of values obtained from counting duplicate in three independent experiments. (** *p* < 0.01 and *** *p* < 0.001 regarded as significant difference).

Next, synergistic anti-RSV effect of PAE and CTE were determined in HEp2 cells. HEp2 cells were infected with RSV-GFP (0.1MOI) for 2 h in DMEM containing 1% FBS. Then cells were treated with PAE, CTE along or as a combination (1:1) in DMEM containing 10% FBS. At 48 hpi GFP absorbance were taken, and the virus titer was determined by standard plaque assay. GFP expression level was significantly reduced with the increasing dose either PAE, CTE along or combination treatment (Figure 3A). However, there was no significant difference observed in combined herb extract treatment compared to individual herb extract treatment at the same final dose treatment. Furthermore, a similar result was observed in the virus replication quantification by standard plaque assay (Figure 3B). Therefore, this data demonstrates that PAE or CTE does not enhance anti-RSV activity synergistically.

### 3.4. Determination of the Effective Concentration (EC_50_) and Cytotoxic Concentration (CC_50_) of PAE and CTE

EC_50_ values of the herb extracts were determined against RSV on HEp2 cells. For this experiment, a GFP assay was performed with some modifications, as described previously [25,26]. Briefly, RSV-GFP virus was used, and a 50% reduction in GFP expression was considered equivalent to a 50% reduction in virus titer. As shown in Figure 3D,F, PAE and CTE inhibited RSV-GFP infection (MOI, 0.1) by 50% at concentrations of 39.82 μg/mL and 27.95 μg/mL, respectively. Next, we determined the CC_50_ values of the two extracts based on a cell cytotoxicity assay using HEp2 cells. The assay showed CC_50_ values of 938.43 μg/mL and 764.17 μg/mL for PAE and CTE, respectively (Figure 3F,G). Interestingly, the cell viability at the effective concentrations of both extracts was greater than 80%. The selectivity index (SI) indicates the safety of a crude extract against RSV infection [19]. The SIs of PAE and CTE were 23.5 and 27.3, respectively (Figure 3H). This data suggests that both PAE and CTE could be used safely as therapeutic agents against RSV infection.

### 3.5. Effect of PAE and CTE on the Production of Viral RNA and Protein in HEp2 Cells

**Figure 4:**
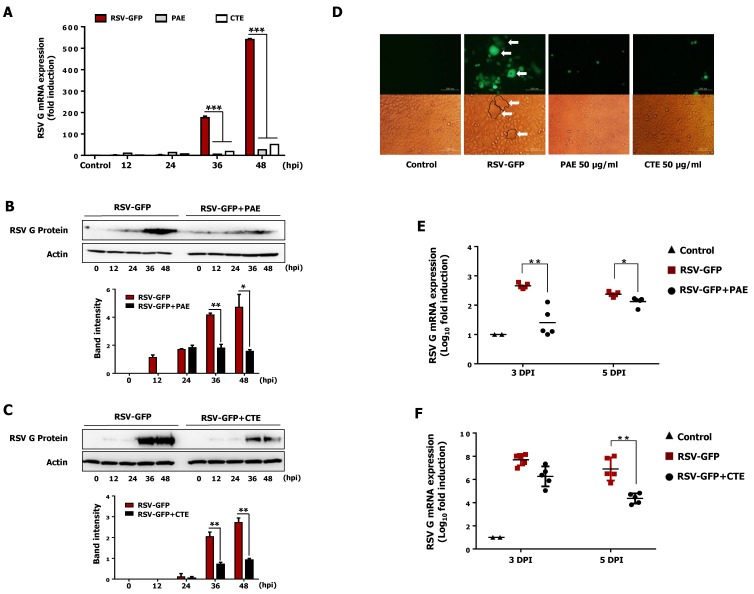
Reduction of RSV Glycoprotein (RSV-G) gene transcription, protein translation and syncytium formation in-vitro and inhibition of RSV replication *in-vivo* by PAE and CTE. HEp2 cells were seeded in six well cell culture plats and incubate for 12 h. Medium was changed into DMEM containing 1% FBS and RSV-GFP (0.1MOI) was infected for 2 h. Then cells were treated with 50 μg/mL PAE or CTE. (**A**) Cells were harvested at indicated time points, and RSV-G protein mRNA level was measured at 12, 24, 36, 48 hpi by qRT-PCR, Glyceraldehyde 3-phosphate dehydrogenase (GAPDH) was used for the normalization. (**B**,**C**) Immunoblot analysis was performed using cell lysates harvested at indicated time points to measure RSV-G protein and β-actin protein expression level time-dependently. The intensity of the RSV-G was quantified. (**D**) Cell and the GFP image were taken at 48 hpi to see the syncytial formation inhibition by PAE and CTE (400× magnification). (**E**,**F**) Five weeks old (16 g/mice) BALB/c mice (*n* = 5) were intranasally infected with RSV-GFP (1 × 10^6^ PFU/mice) in the total volume of 28 μL. PAE or CTE were orally administrated at a dose of 200 μL/mice (0.5 mg/mL) at 6, 12, 18 and 24 hpi. At 3 and 5-day post infection (dpi), lung tissues were collected, and the transcription level of RSV-G protein mRNA was determined by qRT-PCR. The arrow indicates the RSV syncytium formation in HEp2 cells. mRNA expression, band intensity expressed as mean ± SD. Error bars indicate the range of values obtained from three independent experiments. In vivo experiment was performed in duplicate. (* *p* < 0.05, ** *p* < 0.01 and *** *p* < 0.001 regarded as significant difference).

Inhibition of intracellular viral RNA transcription and protein translation by herb extract treatment was evaluated in HEp2 cells. HEp2 cells were infected with RSV-GFP and treated with PAE and CTE (50 μg/mL) at 2 hpi, then cells were harvested at the indicated time points, and viral gene expression at the RNA and protein level was determined by qRT-PCR and immunoblot analysis, respectively. Interestingly, PAE treatment reduced the transcription of RSV-G mRNA level by 39-fold at 36 hpi and 22-fold at 48 hpi. Similarly, CTE treatment reduced the transcription of RSV-G mRNA level by 10-fold at 36 hpi and 11-fold at 48 hpi (Figure 4A). Furthermore, the reduction in viral gene transcription in HEp2 cells treated with herb extracts was associated with reduced RSV-G protein synthesis (Figure 4B,C). Thus, the reduction of viral gene transcription and protein synthesis by PAE and CTE correlated with their antiviral activity in vitro (Figure 2B,C).

### 3.6. PAE and CTE Inhibits RSV Syncytium Formation

Syncytium formation by RSV is a well-known mechanism of cell-to-cell infection that contributes significantly to virus spread in vivo. Therefore, to determine whether PAE and CTE prevent the cell-to-cell spread of the virus after infection, a syncytium formation assay was performed on infected HEp2 cells. Monolayers of HEp2 cells were infected with RSV-GFP and incubated at 37 °C for 2 h. Cells were left untreated or treated with PAE or CTE (50 μg/mL) and examined for syncytium formation. Interestingly, in untreated HEp2 cells, large areas of syncytium formation were visible. By contrast, PAE and CTE treated HEp2 cells showed significantly reduced syncytium formation at 48 hpi (Figure 4D).

### 3.7. Oral Administration of PAE and CTE Enhance Protection against RSV Infection in BALB/c Mice

Next, we designed a mouse model to evaluate the therapeutic effect of PAE and CTE against RSV infection in vivo. BALB/c mice were intranasally infected with RSV-GFP (1 × 10^6^ PFU/mouse) or left uninfected, and PBS, PAE, or CTE were orally administrated at 6, 12, 18, and 24 hpi. The RSV infection titer (1 × 10^6^ PFU/mouse), inoculation time (6, 12, 18, and 24 hpi) and inoculation dose (200 μL (0.5 mg/mL) mice/time were chosen based on preliminary studies. Lungs were collected aseptically at 3 dpi and 5 dpi. RSV-G protein mRNA level in the lungs was determined by qRT-PCR. As shown in Figure 4E, PAE-treated mice showed significantly reduced viral mRNA in the respiratory tract at both 3 dpi and 5 dpi compared to the control (PBS)-treated group. Similarly, the transcription of RSV-G mRNA was reduced in the CTE-treated group compared to the PBS-treated group at 3 dpi and 5 dpi, and was significantly reduced at 5 dpi (Figure 4F). This data demonstrates that both herb extracts have the ability to inhibit viral replication in the mouse respiratory tract and protect against RSV infection in vivo.

### 3.8. Acteoside Inhibits RSV Replication at Non-Cytotoxic Concentrations in vitro

**Figure 5:**
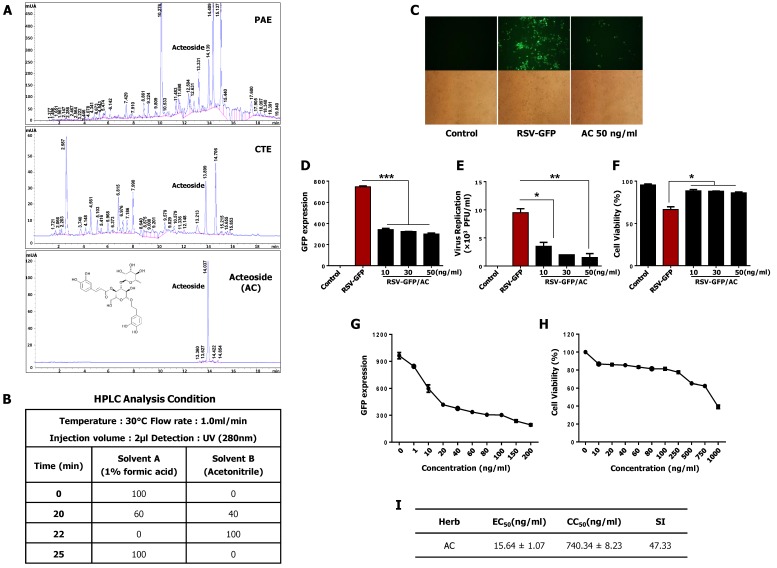
Identification and antiviral effect of acteoside (AC) *in-vitro.* (**A**,**B**) Chemical compounds in PAE and CTE were analyzed by the reversed phase HPLC. The monolayer of HEp2 cells was infected with RSV-GFP (0.1MOI) for 2 h with DMEM containing 1% FBS. Then, the medium was replaced with DMEM containing 10% FBS and cells were treated with 10, 30, 50 (ng/mL) AC. (**C**) After 48 h, images were obtained (200× magnification). (**D**) GFP absorbance levels were measured by Gloma multi-detection luminometer (Promega). (**E**) Viruses were titrated from the cell supernatant and cells by standard plaque assay. (**F**) Cell viability was determined by trypan blue exclusion assay at 48 hpi. (**A**) HEp2 cells were infected with RSV-GFP (O.1MOI) for 2 h with DMEM containing 1% FBS. Then, the medium was changed to DMEM containing 10% FBS and cells were treated with various concentrations AC. 48 hpi GFP expression level was determined. (**G**) HEp2 cells were treated with various concentrations of AC and cell viability was determined at 48 hpi by cell cytotoxicity assay kit. (**H**) To calculate EC_50_ value, 50% reduction of GFP expression was considered as equivalent to the 50% reduction in virus titer. (**I**) The ratio between CC_50_ and EC_50_ considered as Selectivity Index (SI). GFP absorbance, cell viability and virus titer expressed as mean ± SD. Error bars indicate the range of values obtained from counting duplicate in three independent experiments. In vivo experiment was performed in duplicate. (* *p* < 0.05, ** *p* < 0.01 and *** *p* < 0.001 regarded as significant difference).

**Figure 6:**
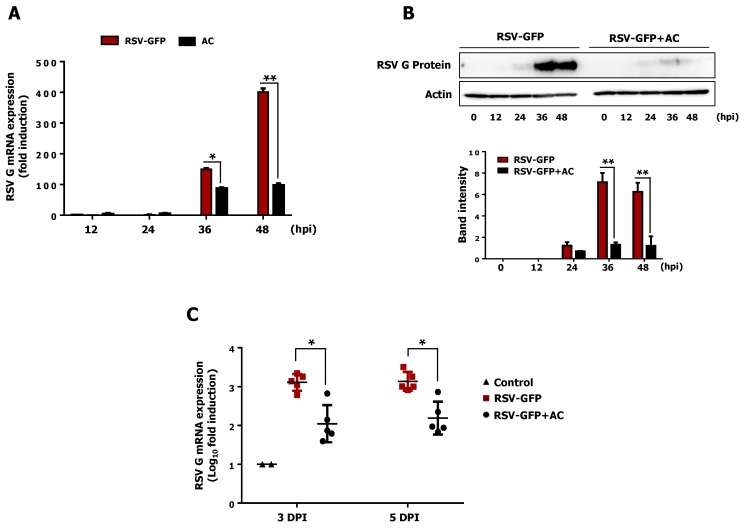
Antiviral effect of acteoside *in-vitro* and *in-vivo*. (**A**) RSV-GFP infected cells were treated with 50 ng/mL of AC at 2 hpi and cells were harvested at indicated time points. RSV-G protein mRNA transcription level was determined by qRT-PCR. GAPDH was used for normalization. (**B**) Infected cells were treated with 50 ng/mL concentration of AC at 2 hpi and cells were harvested at indicated time points. RSV-G protein expression was determined by immunoblotting with anti-RSV-G protein antibody, and the intensity of the RSV-G was quantified. (**C**) Five weeks old (16 g/mice) BALB/c mice (*n* = 5) were intranasally infected with RSV-GFP (1 × 10^6^ PFU/mice) in the total volume of 28 μL. 6 hpi AC was intraperitoneally administrated at a dose of 80 mg/Kg body weight of mice. At 3 and 5 dpi, lung tissues were collected, and the transcription level of RSV-G protein mRNA was determined by qRT-PCR. mRNA expression and band intensity expressed as mean ± SD. Error bars indicate the range of values obtained from three independent experiments. In vivo experiment was performed in duplicate. (* *p* < 0.05 and ** *p* < 0.01 regarded as significant difference).

It has been previously reported that acteoside is an important phenolic glycoside in PAE [27] and CTE [15,17]. To investigate for the presence of this glycoside in PAE and CTE, we performed reverse-phase high-performance liquid chromatography (HPLC) on extracts from PAE and CTE. Interestingly, we found that both extracts contained acteoside as one of their major active components (Figure 5A). To assess the antiviral effect of acteoside, a monolayer of HEp2 cells was infected with RSV-GFP, and at 2 hpi, cells were treated with 10, 30, or 50 ng/mL acteoside and virus replication were monitored. As reported in Figure 5C,D, acteoside treatment significantly reduced the GFP expression compared to untreated HEp2 cells. Furthermore, virus titers were determined by plaque formation assay. Similar to the results of the GFP expression analysis, acteoside significantly reduced the RSV plaque titer (Figure 5E). Cell death induced by RSV infection was also reduced in acteoside-treated HEp2 cells compared with untreated cells (Figure 5F). Next, the EC_50_ and CC_50_ of acteoside were determined in HEp2 cells as 15.64 ± 1.07 ng/mL and 740.34 ± 8.23 ng/mL, respectively (Figure 5G,H). The SI of acteoside against RSV-GFP was 47.33 (Figure 5I). Based on this data, the reduced viral replication caused by actioside (Figure 5C–F) was due to its antiviral properties and not its cytotoxicity. Moreover, the mRNA and protein expression of viral genes in HEp2 cells treated with acteoside was determined, as with the herb extracts. Interestingly, acteoside-treated cells showed significantly reduced RSV-G mRNA compared with untreated cells (1.7-fold reduction at 36 hpi and 4-fold reduction at 48 hpi) (Figure 6A). Similarly, RSV-G protein synthesis was inhibited by acteoside treatment (Figure 6B). Therefore, our results demonstrate that acteoside can inhibit RSV replication in HEp2 cells.

### 3.9. Intraperitoneal Administration of Acteoside Inhibits RSV Infection In Vivo

To further evaluate the therapeutic effect of acteoside against RSV, we investigated its antiviral activity in BALB/c mice. Mice were intranasally infected with RSV-GFP (1 × 10^6^ PFU/mouse), and acteoside was administrated intraperitoneally at 6 hpi at a dose of 80 mg/kg, as described previously [28]. Lungs from all mice were collected at 3 dpi and 5 dpi, and RSV-G mRNA was measured by qRT-PCR. As shown in Figure 6C, the level of viral mRNA was significantly lower in the acteoside-treated group than in the PBS control group at both 3 dpi and 5 dpi. When taken together, acteoside exhibited a strong in vivo antiviral effect and protected mice from RSV infection.

## 4. Discussion

Traditional medicines have been used as remedies against infectious diseases for thousands of years, due to their significant anti-inflammatory, anti-microbial activity and low rate of adverse effects [29,30]. These medicines have been gaining in popularity, due to concerns related to the side effects, high cost, and lack of efficacy of conventional Western medicines [31]. In the years 2001 and 2002, approximately one-quarter of the bestselling drugs worldwide were natural products or were derived from natural products [32]. Recent publications show that traditional Chinese medicinal herbs account for 10% of the prescription drugs in China. They are perceived as harmless and natural and are widely used in many parts of the world, individually or in combination [33]. In particular, medicinal plants have shown potential therapeutic effects against a wide range of respiratory tract-related viral infections, including Severe Acute Respiratory Syndrome (SARS) [34,35,36], Influenza [37,38], and RSV [26,27,39]. Among the thousands of promising medicinal herbs, *Plantago asiatica* and *Clerodendrum trichotomum* are well-known and commonly used in traditional medicine in China, Japan, and South Korea [9,14,15].

In the present study, we screened a library of herb extracts to identify novel therapeutic inhibitors of RSV infection. Interestingly, we identified both *Plantago asiatica* extract (PAE) and *Clerodendrum trichotomum* extract (CTE) as hits with potent antiviral effects against RSV infection in HEp2 cells (Supplementary Figures S1 and S2). Next, we confirmed the dose-dependent anti-RSV activity of both herbs in the HEp2 cell line in detail (Figure 1 and Figure 2).

In addition, the CC_50_ values of PAE and CTE were several magnitudes higher than the EC_50_ values (Figure 3), which is consistent with a favorable safety profile. Furthermore, both herb extracts reduced intracellular viral gene transcription and protein synthesis in vitro, and oral administration of the herbs to infected mice significantly reduced viral gene transcription in the lungs (Figure 4). Finally, we found that acteoside, a common phenolic glycoside present in both herb extracts, is involved in the antiviral activity of the herbs against RSV infection (Figure 5 and Figure 6).

PAE and CTE significantly reduced RSV replication and RSV-mediated syncytial formation in the HEp2 cell line in a dose-dependent manner (Figure 1C). Moreover, we were curious to evaluate whether PAE and CTE can work synergistically to enhance anti-RSV activity. However, herb extracts did not show synergistic anti-RSV effect compared to the individual treatment at the same dose (Figure 3A,B). Since, both extracts reduce the virus replication at the same level when treated alone, or together, at same dose (Ex: PAE, 50 μg/mL and CTE, 50 μg/mL or PAE, 25 μg/mL +CTE, 25 μg/mL), it’s possible that both herb extracts undergo their own mechanism of action for anti-RSV function other than work synergistically.

However, PAE and CTE did not show any cytotoxic effect in the HEp2 cell line (Figure 3F,G). The CC_50_ values of PAE and CTE were 938.43 μg/mL and 764.17 μg/mL, respectively, which were several magnitudes higher than EC_50_ values of 39.82 μg/mL and 27.95 μg/mL, respectively. Even though both extracts were used at a concentration of 50 μg/mL for in vitro experiments, their high SI indicates a broad safety margin for therapeutic purposes.

To support the observation mentioned above, we investigated the intracellular RSV gene transcription in infected epithelial cells. Interestingly, PAE and CTE-treated HEp2 cells showed significantly reduced RSV-G mRNA at 36 and 48 hpi (Figure 4A). This reduction in viral mRNA transcription positively correlated with the low level of viral replication observed at the same time points (Figure 2A,B). Furthermore, PAE- and CTE-treated HEp2 cells showed significantly reduced RSV-G protein synthesis at late time points after virus infection (Figure 4B,C). Therefore, it is possible that PAE and CTE may affect the replication of RSV not only at the transcriptional level, but also at the posttranscriptional level. Syncytium formation by RSV is a well-known mechanism of cell-to-cell infection that contributes significantly to virus spread in vivo. PAE and CTE also significantly reduced the RSV-dependent formation of syncytia in HEp2 cells (Figure 4D). Since both herb extracts could inhibit RSV replication in vitro, we went on to test their antiviral potential in vivo. In vivo replication of RSV can also be accurately assessed by qRT-PCR [40].

Interestingly, our in vivo results (Figure 4E,F) revealed that oral administration of PAE and CTE significantly reduced the amount of RSV-G transcription in the lung at 3 and 5 dpi. These in vivo results are consistent with the low virus titers observed in HEp2 cells treated with PAE and CTE in vitro. Even though the exact underling therapeutic anti-RSV mechanisms of PAE and CTE are still under investigation, our results of the in vitro and in vitro antiviral assays show that both herb extracts significantly abolished cell to cell virus infection of RSV, and ultimate inhibition of virus spreading in the infected sites.

Phenylethanoid glycosides are widely found in edible plants and foodstuffs derived from plants [41,42], and these compounds have numerous biological properties, including anti-hepatotoxic [43], anti-inflammatory, anti-nociceptive [44], and anti-oxidant [45] effects. Phenylethanoid glycosides are one of the major bioactive constituents present in both *Plantago asiatica* [46] and *Clerodendrum trichotomum* [47]. HPLC analysis was conducted to confirm this finding (Figure 5A), and acteoside was identified as one of the major constituents of both PAE and CTE. Acteoside, also called kusagin or verbascoside [48], is a phenylethanoid glycoside isolated from many dicotyledons. Reportedly, acteoside has anti-oxidant and anti-inflammatory properties, and prevents cell apoptosis [39,49]. A recent study demonstrated that acteoside induced ERK activation and subsequent IFN-γ production; thus, showed antiviral activity against influenza and vesicular stomatitis virus (VSV) [28]. Based on these reported findings, we evaluated the antiviral effect of acteoside against RSV. Interestingly, acteoside reduced RSV replication and virus-induced cell death in HEp2 cells (Figure 5E,F) similar to PAE or CTE treatment. In addition, the SI of acteoside against RSV in vitro indicates a high safety margin for its therapeutic effect (Figure 5I). We also confirmed that acteoside reduced the level of RSV-G mRNA and RSV-G protein synthesis at 36 and 48 hpi (Figure 6A). Specifically, intraperitoneal treatment with acteoside showed an anti-RSV effect in a mouse model (Figure 6C). These results are similar to the antiviral activity of acteoside against Influenza or VSV [28]. However, further studies demonstrating a detailed mechanism of how acteoside inhibit RSV replication are needed.

In conclusion, the favorable safety profile and antiviral activity of PAE, CTE, and acteoside suggest that both herb extracts may be good candidates for antiviral therapy for RSV infection. Thus, oral administration of *Plantago asiatica* and *Clerodendrum trichotomum* or administration of acteoside could have potential therapeutic applications in both humans and livestock.

## Supplementary Materials

The following are available online at https://www.mdpi.com/1999-4915/11/7/604/s1, Figure S1. Herb extract library screening results, Figure S2. Selected herb extracts second round screening results.
