# Supplementary material for: Anti-Respiratory Syncytial Virus Activity of Plantago asiatica and Clerodendrum trichotomum Extracts In Vitro and In Vivo
Source: Viruses. 2019 Jul 3;11(7):604. doi: 10.3390/v11070604 (PMC6669655; doi:10.3390/v11070604)
Supplement: Supplementary file 1 [file viruses-11-00604-s001.pdf]

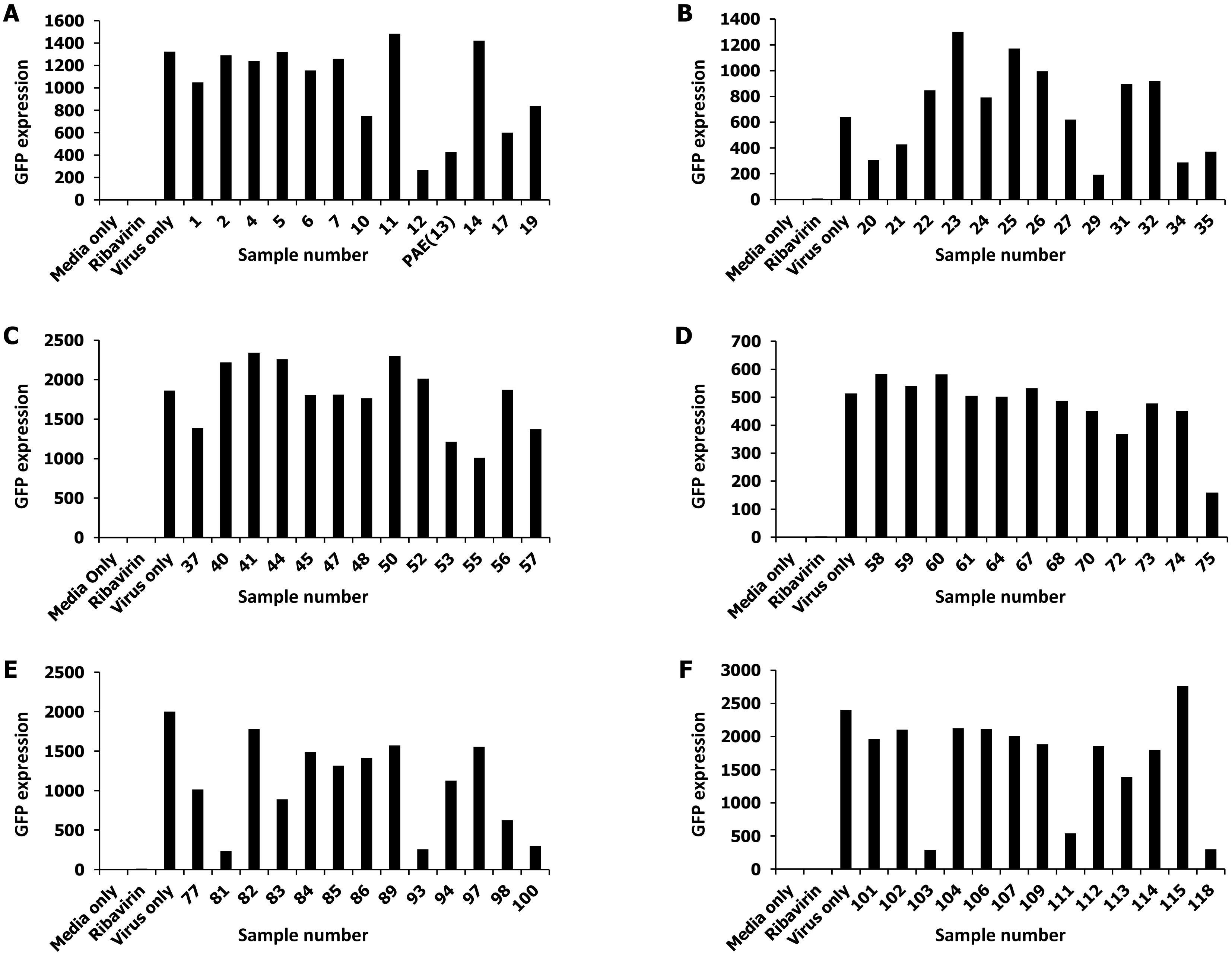
A
B
C
D
E
F
GFP expression
Sample number
Media only
Media Only
Ribavirin
Virus only
1 2 4 5 6 7 10 11 12 PAE(13) 14 17 19
20 21 22 23 24 25 26 27 29 31 32 34 35
37 40 41 44 45 47 48 50 52 53 55 56 57
58 59 60 61 64 67 68 70 72 73 74 75
77 81 82 83 84 85 86 89 93 94 97 98 100
101 102 103 104 106 107 109 111 112 113 114 115 118

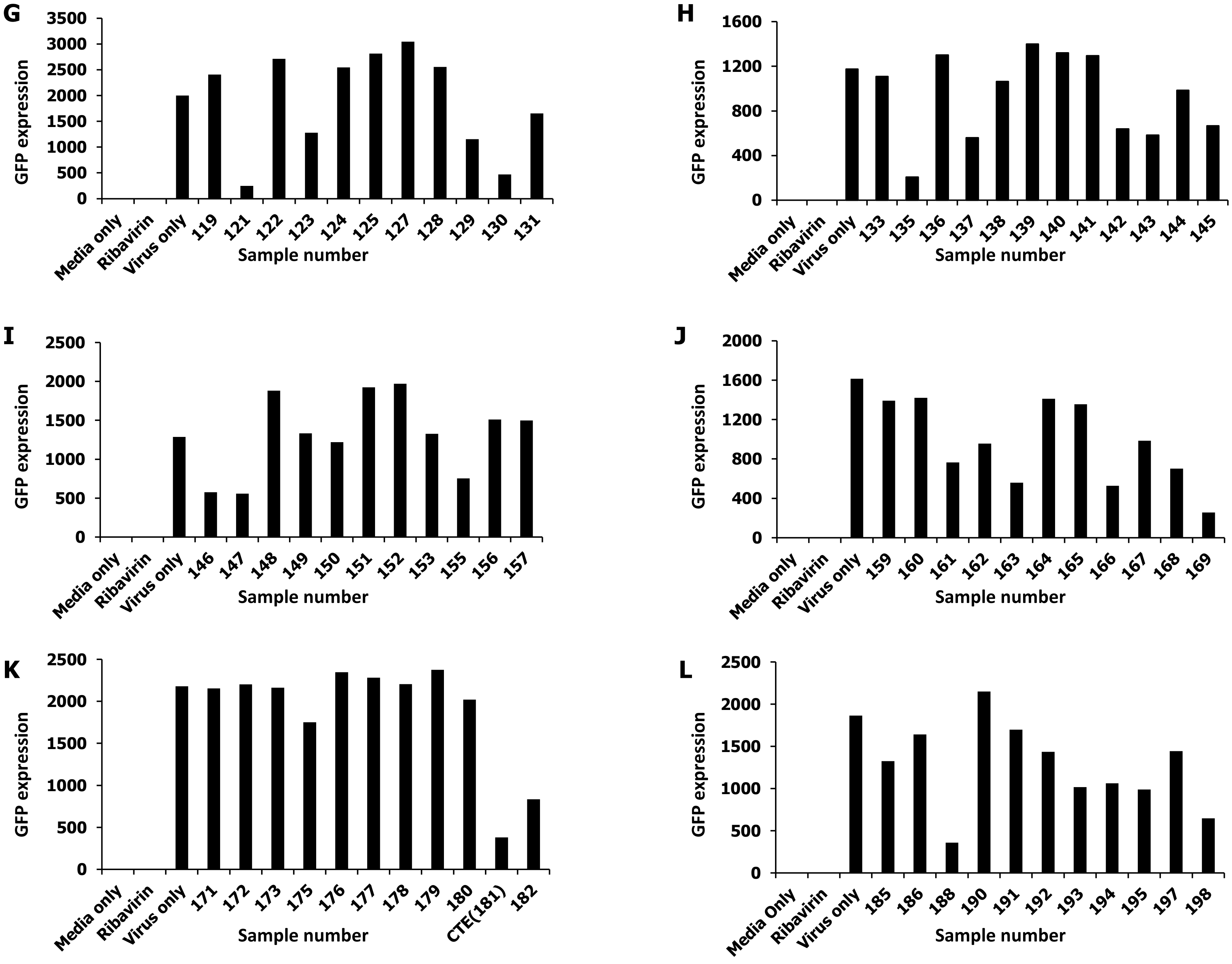

G
GFP expression
3500
3000
2500
2000
1500
1000
500
0
Media only
Ribavirin
Virus only
119
121
122
123
124
125
127
128
129
130
131
Sample number
H
GFP expression
1600
1200
800
400
0
Media only
Ribavirin
Virus only
133
135
136
137
138
139
140
141
142
143
144
145
Sample number
I
GFP expression
2500
2000
1500
1000
500
0
Media only
Ribavirin
Virus only
146
147
148
149
150
151
152
153
155
156
157
Sample number
J
GFP expression
2000
1600
1200
800
400
0
Media only
Ribavirin
Virus only
159
160
161
162
163
164
165
166
167
168
169
Sample number
K
GFP expression
2500
2000
1500
1000
500
0
Media only
Ribavirin
Virus only
171
172
173
175
176
177
178
179
180
CTE(181)
182
Sample number
L
GFP expression
2500
2000
1500
1000
500
0
Media Only
Ribavirin
Virus only
185
186
188
190
191
192
193
194
195
197
198
Sample number

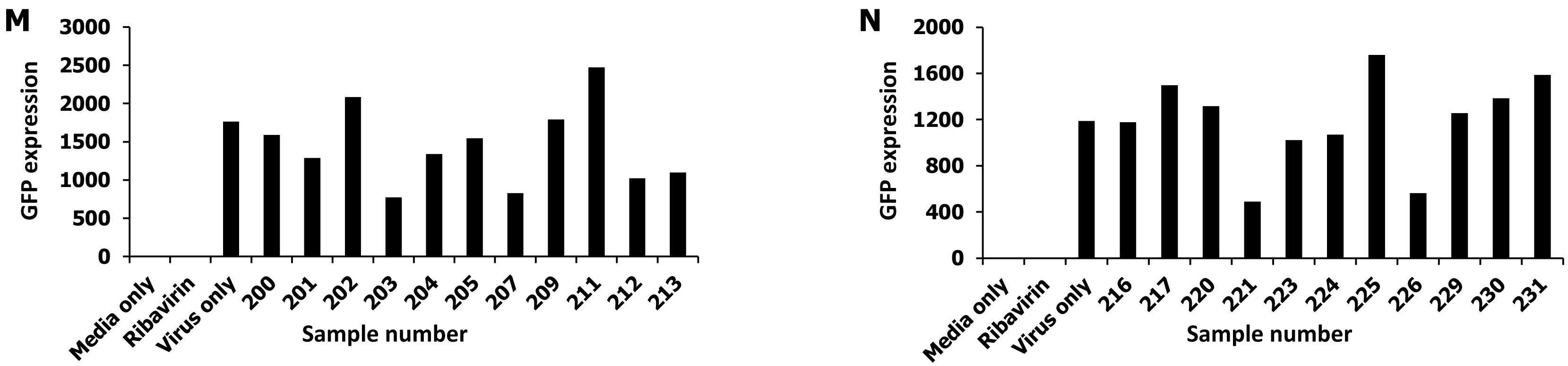

Figure S1. Herb extract library screening results. HEp2 cells were seeded into 96 well tissue culture black plates (SPL) with the cell number of 2.5 × 10^4 cells/well. Twelve hours later, the medium was changed to 1% fetal bovine serum (FBS) containing Dulbecco's Modified Eagle's Medium (DMEM) and cells were infected with RSV-GFP (0.1MOI) or kept uninfected. Two hours later, the medium was replaced with 10% FBS containing DMEM and cells were treated with 30 μg/mL of herb extracts or 30 μM of ribavirin as positive control. Cells without any treatment regard as virus only. (**A-N**) At 48 hpi medium was replaced by autoclave PBS and GFP absorbance levels were measured by Gloma multi-detection luminometer (Promega). Candidates that reduced more than 80% GFP absorbance with respective to virus only treatment of same experiment were selected for second round.

A

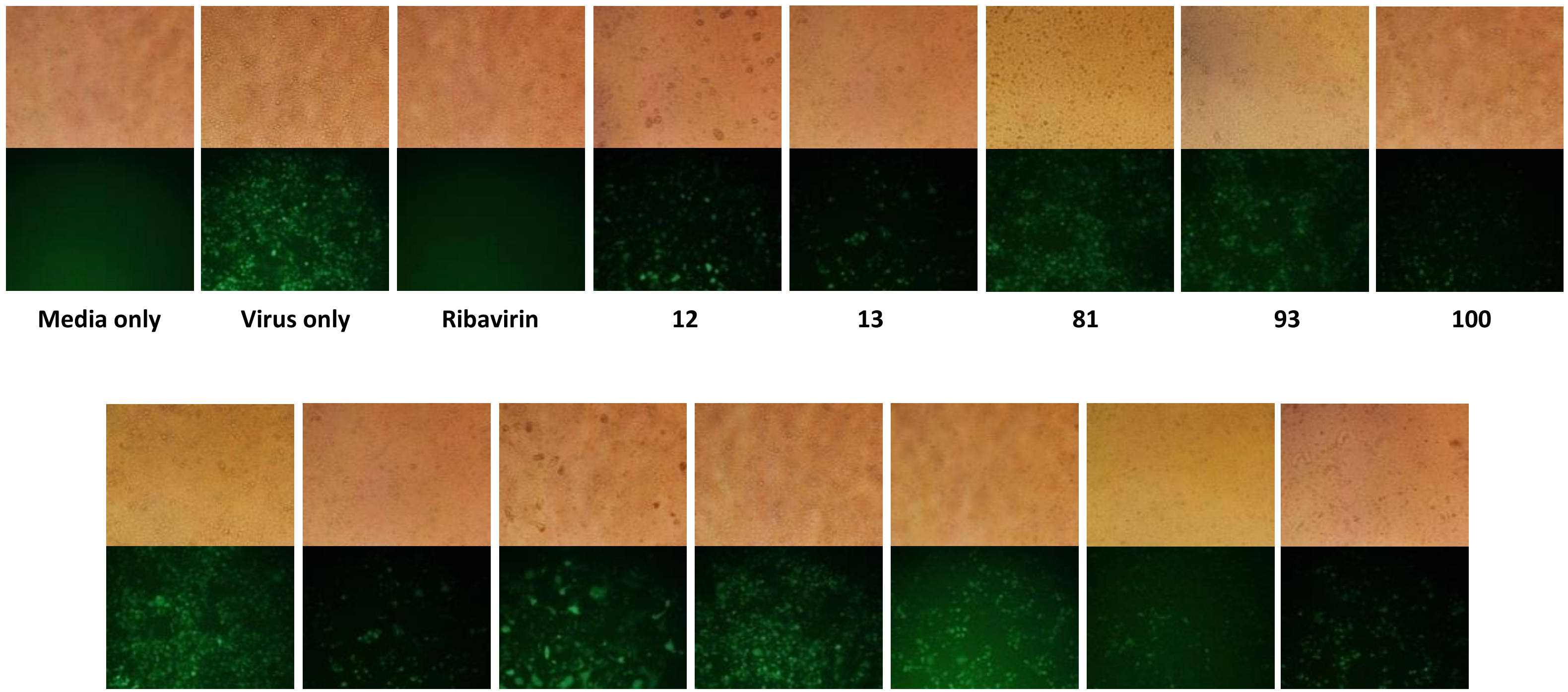

B

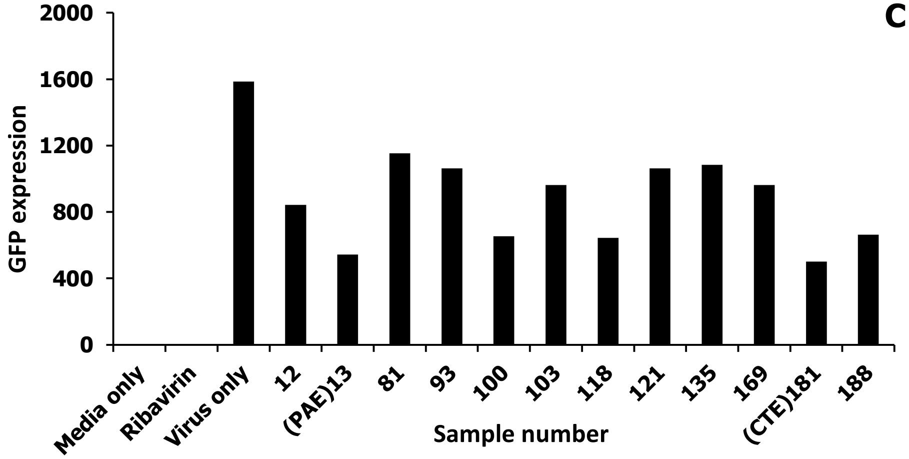

C

Selected samples with anti-RSV effect

Number	Sample number
01 (PAE)	13
02	100
03	118
04 (CTE)	181

Figure S2. Selected herb extracts second round screening results. HEp2 cells were seeded into 12 well cell culture plates with the cell number of 2.5 × 10^5 cells/well. Twelve hours later, the medium was changed to 1% fetal bovine serum (FBS) containing Dulbecco's Modified Eagle's Medium (DMEM) and cells were infected with RSV-GFP (0.1MOI) or kept uninfected. Two hours later, the medium was replaced with 10% FBS containing DMEM and cells were treated with 30 μg/mL of herb extracts or 30 μM of ribavirin as positive control. Cells without any treatment regard as virus only. (**A**) After 48 h, images were obtained (200 × magnification). (**B**) GFP absorbance levels were measured by Gloma multi-detection luminometer (Promega). (**C**) Candidates that reduced GFP absorbance with no cytotoxicity were selected for future experiments (PAE#13, CTE#181).
